# Combined use of xenogenous bone blocks and guided bone regeneration for three-dimensional augmentation of anterior maxillary ridge: A case series

**DOI:** 10.15171/japid.2019.015

**Published:** 2019-12-18

**Authors:** Mohammadreza Talebi, Noushin Janbakhsh

**Affiliations:** ^1^Department of Periodontology, School of Dentistry, Shahid Beheshti University of Medical Sciences, Tehran, Iran; ^2^Department of Periodontology, School of Dentistry, Ilam University of Medical Sciences, Ilam, Iran

**Keywords:** Alveolar bone grafting, alveolar bone loss, Heterograft

## Abstract

**Background:**

Bone augmentation ensures a favorable 3-dimensional position of implants. Onlay grafting is one of the techniques in ridge augmentation, which can be performed with the use of xenogenous blocks.

**Methods:**

Three cases of the vertical and horizontal ridge are discussed, which were augmented using xenogenous blocks. The blocks were shaped in a favorable size and puzzled along the grafting area. All the gaps were filled with granular xenografts. The flaps were coronally advanced to obtain primary closure.

**Results:**

An average of 4.2-mm gain in width and 4.2-mm gain in height of the ridge was observed at the implantation stage.

**Conclusion:**

The outcomes of these cases could pave the way for suggesting xenograft blocks for augmenting wide areas of the alveolar ridge on average of 4 mm in width and height in selected cases as an alternative to standard autogenous blocks. Long-lasting xenograft ensures implant and lip support in the esthetic zone.

## Introduction

Several materials and techniques have been developed to augment the alveolar bone.^1^ Autogenous bone as the gold standard graft material has several disadvantages, including morbidity of the donor site, patient discomfort, unpredictable resorption of the graft, and the limited quantity.^2^ The use of xenografts has been advocated due to the lack of the shortcomings mentioned above.^3^

Various techniques of ridge augmentation include guided bone regeneration, onlay/veneer grafts, inlay grafts, distraction osteogenesis, and ridge splitting.^1,4^ Onlay xenografts are reported to result in 97.1% implant survival.^4^ In this technical note, we present three cases of vertical and horizontal ridge augmentation using xenogenous bone blocks.

## Methods

**Figure 1:**
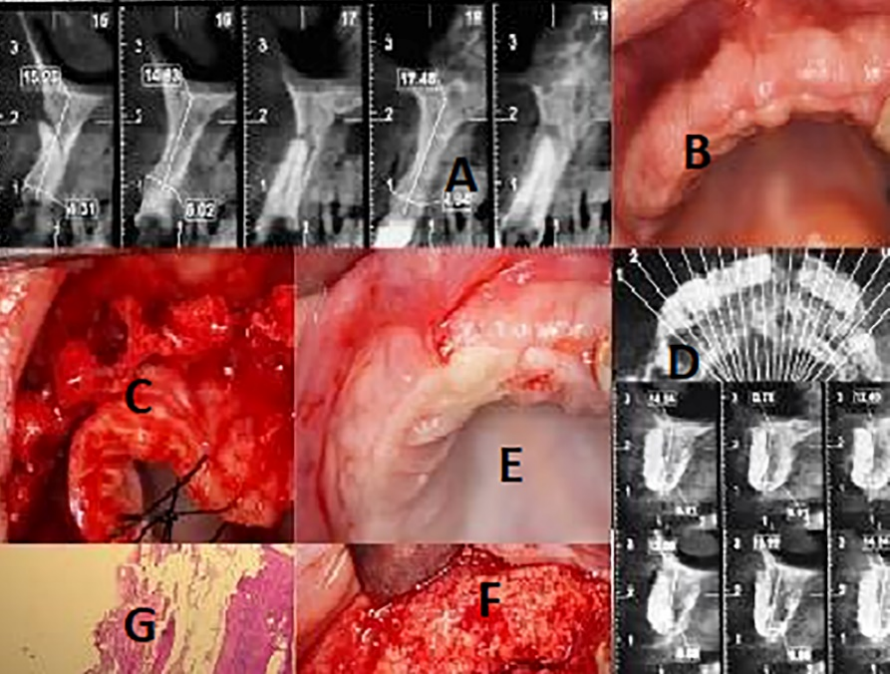
Case 1: 39-year-old female. a) The initial CBCT at the augmentation phase showing very poor dimensions of the bone (due to the previous traumatic extractions). b) The ridge at the augmentation session. c) Residual ridge following full-thickness flap elevation. d) CBCT of the patient 9 months later shows block integration. e) Clinical view at the implantation session, adequate height, and width of the bone and complete integration of the graft. f) Full-thickness flap. g) While preparing the implant site a trephine biopsy was obtained from the site #16. The biopsy specimen stained by hematoxylin-and-eosin and magnified ×200 shows newly formed bone at the site of implant placement and remnants of the xenograft. Although more remnants and connective tissue might be present at the buccal side, it is of importance that in the favorable site of implant placement only native bone is present.

**Figure 2:**
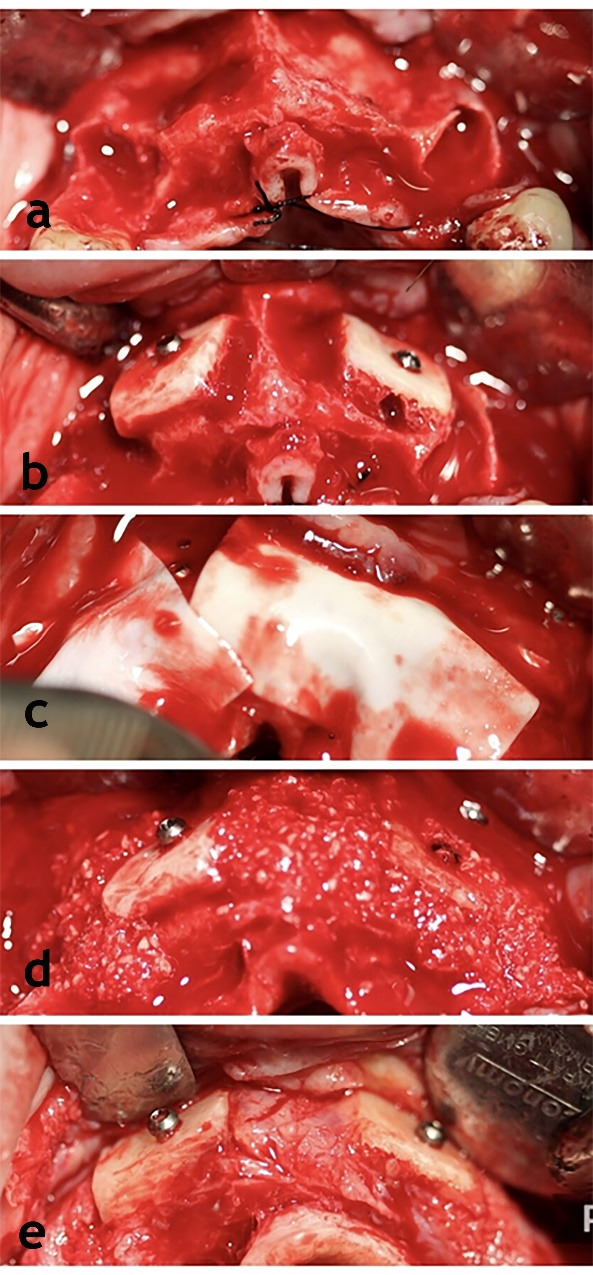
Case 2 was a 51-year-old female. a) Less traumatic extraction of hopeless teeth. b) Block fixation using fixation screws; depending on the size of the blocks used one screw could provide enough fixation. c) Particulate xenograft fills the space between the block grafts. d) A double-layered membrane is fixed. e) At the implantation session, a full-thickness flap was raised, revealing complete integration of the grafts and adequate dimensions of the ridge for implant placement.

Three consecutive generally healthy patients in a private periodontal office with deficient maxillary alveolar ridge <3 mm in thickness buccolingually were included (Figures 1‒2). The primary defect size and location were assessed using CBCT images (Table 1).

**Table 1:** The overview of surgical sites augmented by onlay xenografts

**Patient**	**Age**	**M/F**	**Ridge augmentation (xenogenous onlay grafts)**			
			**Graft material**	**Membrane type**	**Grafted area** ^*^	**Healing (months)**
1	39	F	Ceraboneblock-L20†	SIC b-mem (30*40 cm)	4-16	9
2	51	F	Ceraboneblock-L20	SIC b-mem (20*30 cm)	9-13	9
3	50	F	Ceraboneblock-L20	Jason Membrane^‡^ (30*40 cm)	6-14	9

*International Dental Federation tooth-numbering system. ⁋ Healing time before uncovery. † Cerabone block-L20 (20*20*10mm) (aap Biomaterials) ‡ Jason Membrane (30*40cm) (aap Biomaterials)

The patients were pre-medicated with one gr of amoxicillin one hour in advance. A full-thickness flap was elevated by two incisions under local anesthesia: first, an incision on the alveolar crest, which was delicately palatal/lingual; second, a vertical releasing incision on the second tooth away from the surgical site. An incision was also made through the periosteum with a scalpel blade and Medzenbach scissors by continuously opening the scissors and cutting through the tissue attachments partially to allow coronal advancement. The advancement continued until a 2-mm overlap of the buccal flap on the palatal side was observed. Using fine tissue forceps to stretch the buccal flap to overlap the palatal side, the buccal flap should have stayed over the palatal flap to indicate that it was tension-free. However, if the buccal flap started to retract to the buccal side, periosteal releasing was necessary to proceed.

After granulation tissue removal, the recipient site was decorticated thoroughly by a #2 or #4 round bur with a 2-mm distance between the perforations. The resorbable membrane (Table 1) was fixed labially. A xenogenous bone block (Table 1) was molded in pieces measuring about 10 mm in length, 4 mm in width, and 3-4 mm in depth, using the saw tip of a piezosurgery tool. All the pieces were adapted along the grafting area on the buccal side, and each was fixed with one or two screws (8 or 10 mm long). All the gaps between the block pieces were filled using xenogenic particulate bone graft (Cerabone, Bottis, Germany) until slightly over-contoured. After fixing another membrane palatally/lingually, the flap was sutured first by horizontal mattress sutures and then by interrupted sutures in between. It is suggested that this suture be placed in the mucogingival area to help approximate the flaps.

The regimen of 0.2% chlorhexidine mouthwash twice a day was administered for one week, accompanied by amoxicillin (500 mg) three times a day. Ibuprofen (400 mg) was prescribed every 6 hours until the pain was relieved.

The patients were examined the day after surgery, and every 48 hours, to check and render professional cleaning. No complications, including dehiscence and infection of the surgical site, occurred during the follow-ups.

Interrupted sutures were removed by the second week, but the horizontal mattresses remained for one more week. The follow-ups continued monthly. After a healing period of 9 months, a second CBCT was taken to determine the implant size for the second-stage surgery and measurement of new bone formation.

After raising a full-thickness flap, the implants were inserted at an insertion torque of 20 N.cm and submerged for three months until they were uncovered for the prosthetic stage (Table 2). At the time of implantation, bone biopsies were gathered using a trephine bur and assessed histologically to determine graft integration.

**Table 2:** The overview of implant sites and characteristics

**Number of implants**	**Site** ^*^	**Type**	**Length**	**Diameter**
6	4,6,8 9,11,13	Tixos-MC	10 11.5	4.5
4	6, 7, 10, 11	zimmer	10	4.1
5	7, 10, 11, 12, 14	Tixos-MC	10	4.5 3.75

*International Dental Federation tooth-numbering system.

## Results

Table 3 shows the summary of the outcomes at nine months. The new bone appeared well integrated to the recipient site on CBCT images. The results of histological evaluations showed that the xenografts were integrated into the newly formed bone (Figure 1, F‒G). No bone loss, peri-implant mucositis, or implant mobility were recorded.

**Table 3:** The overview of the results: bone gain (millimeters) from CBCT tomography at the time of implantation

**Patient**	**Last follow up** **(months)** ^*^	**Average Width**		**Average Height**	
		**Before augmentation**	**After augmentation**	**Before augmentation**	**After augmentation**
1	12	2.3	6.3	9	13.9
2	24	1.5	6.1	6.7	10.4
3	6	6.5	10.9	1.5	5.5
**Mean**	14	3.4	7.8	5.7	9.9

^*^The last visit after implantation by months.

## Discussion

Xenogenous bone blocks were used to augment extensive horizontal and vertical alveolar ridge defects while managing two of the most common complications associated with them. First, the vertical releasing and periosteal incisions were made to attain tension-free primary closure. Second, piezo-surgery was used to shape the xenograft, which prevented the fracture of the fragile material.

CBCT examinations showed adequate bone gain (average: 4.4 mm horizontally and 4.2 mm vertically). Histologic evaluations showed newly formed bone at the implant site after nine months. Sufficient bone was present at the implantation site to place the implants at the ideal site. The results reported by the sixth European Workshop on Periodontology declared 4.2‒4.6 mm of increase in the vertical height of ridge after autogenous onlay grafting.^5^ Previous studies have reported different amounts of augmentation and xenograft integration in humans, as summarized in Table 4.^6-12^

**Table 4:** A summary of some human studies using xenograft for alveolar bone augmentation

**Authors**	**No. of patients**	**Graft healing (months)**	**Augmentation material**	**Horizontal bone gain**	**Vertical bone gain**	**Graft integration**
Simion et al.^6^	7	3.5	Autog P+ Xeno P+ Ti reinforced e-PTFE mem.	-	3.15 mm	8.63% remaining xeno
Scarano et al.^7^	9	4	Xeno miniblocks + CCPB particles	-	7.43 – 6.68 mm	33% remaining graft
Simion et al.^8^	2	5	Xeno B/P+ rh-PDGF +/- collagen mem.	-	3 mm (with mem) 8 mm (without mem)	Xeno embedded in bone
Proussaefs and Lozada^9^	12	5	Autog B+ Xeno P	-	5.8 mm	23.89% remaining Xeno
Friedmann et al.^10^	28	7	Xeno P+ reorbable or nonresorbable mem	-	-	14 – 15% remaining Xeno
Von Arx and Buser^11^	42	5.8	Autog B+ Xeno P+ collagen mem	4.6 mm		Xeno particles showed either fibrous encapsulation or new bone integration
Hammerle et al.^12^	12	9.5	Xeno B/P	3.6 mm		Xeno integrated into new bone but on the surface of the new bone, only some single xeno particles were integrated.

Xeno: Xenograft; Autog: Autogenous; P: particles; B: block; Ti: Titanium; e-PTFE: expanded-polytetrafluoroethylene; mem: membrane; CCPB: cortico-cancellous porcine bone; +/-: with or without

In this study, growth factors were not used; thus, the use of double-layered resorbable membranes provided a barrier during graft remodeling. This, of course, required a longer period of healing.^13^ Patients also prefer bone substitutes rather than autogenous bone.^1^

The PASS principles, described by Wang and Boyapati^14^ for GBR, can be modified by adding the factor of ‘time’ and be applied for xenogenic block grafting. The primary closure should be obtained through releasing incisions (both vertical and periosteal) and mattress and interrupted sutures. Angiogenesis is obtained by decortication of the bone. Stability would be possible by using fixation screws for blocks. The blocks themselves will provide a tenting effect for the GBR arrears,^15^ and finally, double-layering and screw-fixing of the membrane will provide the complex with stability. Space maintenance is achieved by the use of blocks and particulate bone grafts. Finally comes the time factor, which we believe is the key to the success of the use of xenogenic blocks. A minimum of 9 months is required for the integration and tissue maturation in the augmented site.

Although favorable results were obtained here, augmentation procedures have high morbidity and are very skill-sensitive techniques. Clinical trials with large sample sizes are needed to confirm the results of this study.

## Conclusion

Although autogenous blocks remain the standard, wide areas of bone augmentation were achieved in these cases. Primary closure, angiogenesis, stability, space maintenance, and increased healing time are the keys to successful management. The primary limitation is technique sensitivity.

## Competing Interests

The authors declare no conflict(s) of interest related to the publication of this work.

## Authors’ Contributions

Clinical work, follow ups, design of article and intellect.

## Ethics Approval

None.
